# Body mass index trajectory patterns and changes in visceral fat and glucose metabolism before the onset of type 2 diabetes

**DOI:** 10.1038/srep43521

**Published:** 2017-03-07

**Authors:** Keisuke Kuwahara, Toru Honda, Tohru Nakagawa, Shuichiro Yamamoto, Takeshi Hayashi, Tetsuya Mizoue

**Affiliations:** 1 National Center for Global Health and Medicine, Bureau of International Health Cooperation, Department of Epidemiology and Prevention, Shinjuku-ku, 162-8655, Japan; 2 Teikyo University Graduate School of Public Health, Itabashi-ku, 173-8605, Japan; 3 Hitachi Health Care Center, Hitachi., Ltd., Hitachi, 317-0076, Japan

## Abstract

We investigated BMI trajectory patterns before diabetes diagnosis and examined associated changes in visceral adiposity and glucose metabolism. 23,978 non-diabetic Japanese participants (2,789 women) aged 30–64 years were assessed with a mean follow-up of 7.6 years. Diabetes was diagnosed via fasting glucose, HbA_1c_, and self-report. Latent-class trajectory analyses were performed to identify BMI trajectories. Longitudinal changes in BMI, visceral adiposity, and glucose metabolism were estimated using mixed models. 1,892 individuals developed diabetes. Three distinct BMI trajectories were identified in adults developing and not developing diabetes, respectively. Among adults developing diabetes, 47.3% were classified as “medium BMI” (n = 895), and had increased mean BMI within the obesity category before diagnosis. The “low BMI” group (38.4%, n = 726) had an initial mean BMI of 21.9 kg/m^2^, and demonstrated small weight gain. The “high BMI” group (n = 271) were severely obese and showed greater increase in BMI until diagnosis. All groups which developed diabetes showed absolute and/or relative increase in visceral fat and impaired β-cell compensation for insulin resistance. All groups not developing diabetes showed measured variables were relatively stable during observation. These data suggest that visceral fat gain may induce β-cell failure in compensation for insulin resistance, resulting in diabetes regardless of obesity level.

Diabetes is of growing concern worldwide. In particular, East Asia is experiencing a rapidly emerging diabetes epidemic and accounts for more than 25% of the global diabetic population^1^. Obesity is a major risk factor for type 2 diabetes^2^. However, accumulating evidence has shown that East Asians develop diabetes at a lower degree of obesity than Caucasians^3^, suggesting that the pathogenesis of diabetes may be affected by ethnicity in relation to obesity. Better understanding of ethnic differences in diabetes pathogenesis would contribute to the establishment of ethnically tailored strategies for diabetes prevention and management.

Evidence is limited regarding the development of obesity before the onset of diabetes. Previous studies have assessed average changes in body mass index (BMI) before diabetes diagnosis (i.e., one BMI trajectory within the entire population)^4^,^5^,^6^,^7^,^8^. However, this approach may not be suitable for different ethnic groups where distinct patterns of pathogenesis likely occur. To date, only one study of UK residing Caucasians has assessed BMI trajectory pattern subtypes prior to diabetes diagnosis^9^, all of which were characterized by high BMIs (approximately 25.0 to 40.0 kg/m^2^). In Japanese diabetic patients, mean BMI is approximately 25.0 kg/m^2^ at the first clinical/hospital visit^10^ and the prevalence of obesity (≥25.0 kg/m^2^) does not exceed 50%^11^. Thus, a pattern characterized by low BMI before diabetes diagnosis should be considered in Japan. Additionally, most previous studies have only determined an average change in BMI for adults without diabetes^4^,^5^,^9^. Therefore, it remains unclear how distinct BMI trajectory subtypes can affect the likelihood of diabetes onset. Lastly, trajectory data regarding glucose metabolism, as well as visceral fat, which is more strongly related to glucose metabolism than BMI^12^, will enhance our understanding of the role of obesity in diabetes pathogenesis. Although the aforementioned UK study^9^ provided detailed data on glucose metabolism, visceral adiposity was assessed using only waist circumference.

The primary aim of this study was to investigate BMI trajectory patterns among diabetic patients during the 9 years prior to diabetes onset, and compare them to non-diabetic participants in a Japanese cohort. Secondarily, we aimed to assess longitudinal changes in visceral and subcutaneous fat accumulation measured by computed tomography scans, visceral to subcutaneous fat ratio as an indicator of relative body composition^13^, and markers of insulin resistance, β-cell function, and the ratio of β-cell function to insulin resistance as an insulin disposition index substitute according to the BMI patterns.

## Methods

### Study design

This study assessed a sub-cohort from the Japan Epidemiology Collaboration on Occupational Health (J-ECOH) Study, an on-going, large-scale study of Japanese workers recruited from more than 10 companies in Japan. Details of the J-ECOH Study and the present cohort from one of the participating companies have been described previously^14^,^15^. In Japan, workers are obliged to undergo annual health examinations under the Industrial Safety and Health Act. Workers at the participating companies were informed of the J-ECOH using posters, and were given an opportunity to refuse the use of their data for research. The study protocol was approved by the Ethics Committee of the National Center for Global Health and Medicine, Japan.

The present data were obtained for 42,329 workers (35,378 men and 6,951 women), aged 30 to 64 years, who underwent health check-ups between April 2006 and March 2007. This cohort was assessed independently and their outcomes were reviewed until March 2016. Data regarding visceral and subcutaneous fat, and fasting insulin were extracted from subgroup of the workers with additional measurements of these variables (Supplementary Table S1).

### General health examination

At the health check-up, body height was measured to the nearest 0.1 cm and body weight to the nearest 0.1 kg. BMI was calculated as kg/m^2^. History of disease, work-related factors, and health-related lifestyle factors were ascertained using a standard questionnaire. Details of these measurements have been previously described^15^.

### Body fat and laboratory measurement

Visceral and subcutaneous fat areas (cm^2^) were measured at the umbilical level using computed tomography scans as previously described^16^. Plasma glucose concentrations were assayed using the glucose electrode technique and HbA_1c_ was measured using high-performance liquid chromatography. Serum immunoreactive insulin (μU/mL) was measured using an immunoenzymatic method. Homeostasis model assessment for insulin resistance (HOMA-IR), an index of insulin resistance, and homeostasis model assessment for β-cell function (HOMA-β), an index of β-cell function, were calculated using standard formulas.

### Assessment of type 2 diabetes

Diabetes was identified using data from annual health check-ups for a maximum of 9 years following the first examination. Diabetes was defined as HbA_1c_ ≥ 6.5% (48 mmol/mol), fasting plasma glucose ≥ 7.0 mmol/L (126 mg/dL), or current use of treatment for diabetes. Individuals without diabetes at the first examination who subsequently met any of the above criteria were considered to have an incident case of type 2 diabetes.

### Statistical analysis

Data are shown as mean ± standard deviation for continuous variables or as n (%) for categorical variables. To identify BMI trajectory patterns among individuals who did and did not develop diabetes, respectively, we used latent class trajectory analysis^17^, a type of finite mixture modelling. This analysis is designed to identify distinctive clusters of individuals following similar progressions of outcome over time within a population^18^. We applied a censored normal model^18^ to identify patterns in BMI trajectory. The time from diagnosis of diabetes or last examination was retrospectively used as the time scale (year), ranging from 9 years before diagnosis or examination (−9 years) to the date of diagnosis or last examination (0 years).

We determined the trajectory models regarding the number of groups and trajectory shapes (e.g. linear, quadratic, cubic) based on a two-stage model selection process^19^. At the first stage, to determine the number of groups, we repeated analyses for latent class trajectory by changing the number of groups from two to five, treating BMI as the dependent variable and time before diagnosis or last examination, age, and sex as the independent variables by using the Stata program^18^. We determined the number of groups with consideration of Bayesian information criteria (BICs) as model fit statistics and study objective (identification of low BMI pattern)^19^. After determining the number of groups, we repeated the analyses to determine the trajectory shapes by changing the combinations of trajectory shapes to identify the model showing the best BIC. After determination of the number of groups and trajectory shapes, participants were classified into BMI trajectory groups based on the maximum estimated probability of belonging to each group. Average posterior probability for each group of ≥0.70 is considered as good discrimination in classifying individuals into distinctive groups. We named the BMI groups according to the BMI level (i.e., low, medium, and high) for participants who developed diabetes and those who did not, respectively.

After identification of BMI trajectory patterns for individuals who developed and did not develop diabetes, respectively, longitudinal data on BMI, visceral adiposity, and glucose metabolism according to the BMI groups were retrospectively estimated for each participant from the last to first examination using a linear mixed-effects model, with adjustments for age, sex, and examination year, based on the method of Tabák *et al*.^20^. Pair-wise differences for trajectories during observation period between BMI groups were tested by using an F-test with Bonferroni correction as post hoc analysis in the mixed models. To examine the trend association of participant characteristics at the first examination and BMI groups among adults who developed diabetes and adults who did not, respectively, P values for trend was calculated across BMI groups (from low to high) using logistic or linear regression. Two-sided P values < 0.05 were considered to be statistically significant.

To characterize annual changes in the observed variables by BMI groups statistically, we used linear regression to estimate slope and intercept by treating time as the independent variable and BMI, visceral adiposity, and glucose metabolism as dependent variables for the respective analysis. All analyses were performed using Stata version 14.1 (Stata Corp, College Station, Texas).

## Results

Of the cohort of 42,329 examined participants, 13,059 were excluded at the initial examination in 2006 due to missing data regarding fasting glucose and HbA_1c_ (n = 9,110), diagnosis of diabetes (n = 3,363), or history of ischemic heart disease, stroke, or cancer (n = 766), as these may affect the BMI trajectory. Some participants met two or more exclusion criteria. Of the remaining (n = 29,270), individuals who did not have data on family history, lifestyle, and work-related factors at the first examination were further excluded (n = 2,965). Lastly, we excluded 2,327 workers who did not attend, attended only one subsequent health check-up, or who attended more than one subsequent check-up but had fewer than two BMI measurements. The main analysis included 23,978 workers (21,189 men and 2,789 women), aged 30 to 64 years in 2006 (mean 45.2 years). Although the proportion of women among those remaining in the analysis (n = 23,978) was about 10% lower than that among those who were excluded (n = 18,351), mean levels of age and BMI were not materially different between the excluded and included participants (Supplementary Table S2). Individuals who had data regarding visceral fat during the observation period tended to be male as compared with those who did not have these data, when assessed according to BMI groups (Supplementary Table S3). Mean age and BMI levels were not largely different in any groups.

During the mean follow-up of 7.6 years (182,619 person-years), 1,892 workers developed diabetes. Latent class trajectory analysis showed that, as the number of identified BMI groups increased, BICs were improved in both adults who developed diabetes and in those who did not. BMI trajectory characterized by low BMI was successfully identified when the number of BMI groups was three or more, and an increase in the number of group did not change this finding (identification of low BMI pattern), and limited data on visceral adiposity are available for some groups; therefore, for simplicity and reliability, we decided to use three BMI groups to further subdivide those who developed and those who did not develop diabetes, respectively. The trajectories of BMI and other variables from a model showing the best BIC in the five BMI groups are shown in Supplementary Fig. S1 and Supplementary Fig. S2.

**Figure 1:**
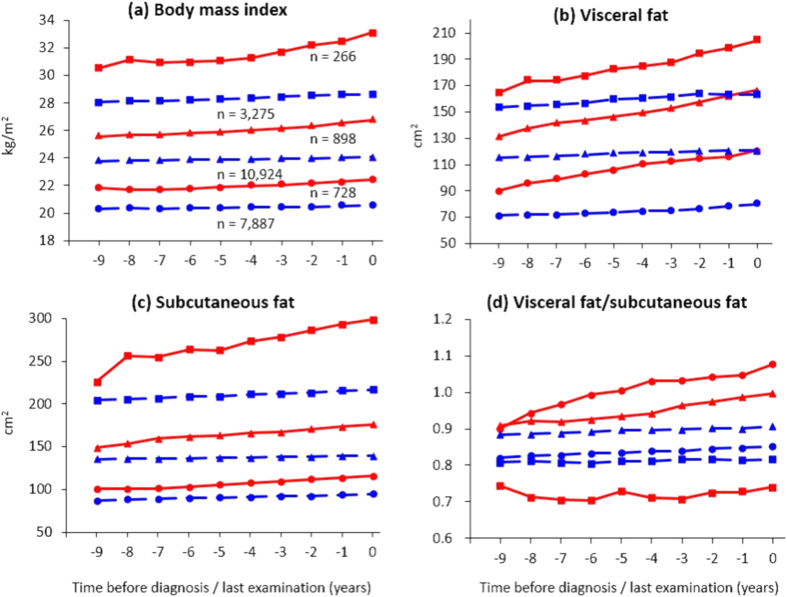
Average changes in BMI, visceral and subcutaneous fat, and ratio of visceral to subcutaneous fat from 9 years before diagnosis/last exam by BMI trajectory. Solid lines in red indicate estimated trajectories for the groups that developed diabetes, while dashed lines in blue indicate trajectories for each group that did not develop diabetes. Square, high BMI; triangle, medium BMI; circle, low BMI.

Among those who developed diabetes, the best BIC for the three BMI trajectory patterns prior to diabetes diagnosis was observed when the shape of trajectory was cubic in the lowest BMI group and quadratic in the two higher BMI groups. Figure 1a illustrates the BMI trajectories. Nearly half of adults who developed diabetes (n = 898, 47.5%) was classified into a group showing mean BMI levels exceeded 25.0 kg/m^2^ over a 10-year period. This group’s mean BMI levels were second highest among individuals who developed diabetes; thus, this group was labelled “medium BMI”. The second largest group (n = 728, 38.5%), labelled “low BMI”, was characterized by consistently low BMI during the follow-up period (medium BMI vs low BMI, P < 0.001). For example, mean BMI was 21.9 kg/m^2^ 9 years before diabetes onset. This group showed a relatively monotonic increase in BMI over time. The third group (n = 266, 14.1%) demonstrated a trajectory characterized by much higher BMI (over 30.0 kg/m^2^) compared with other two groups during the observation period (medium BMI vs high BMI, P < 0.001). Thus, we named this group “high BMI”. Of note, the relative increase in BMI throughout the observation period was 2.5% in the low, 4.8% in medium, and 8.4% in high BMI groups. All groups showed sufficiently high average posterior probability of individuals belonging to each of the groups ( >0.95). The intercept and linear slope for each BMI trajectory are summarized in Table 1.

For adults who did not develop diabetes, when the number of BMI groups was three, the best BIC was detected for the trajectory shape as quadratic for the low and medium BMI groups and as cubic for the high BMI group. These groups showed distinct trajectories of BMI over time (all P < 0.001) and were thus termed as: (1) low BMI (20.3 kg/m^2^ 9 years before the last examination, n = 7,887), (2) medium BMI (23.8 kg/m^2^, n = 10,924), and (3) high BMI (28.1 kg/m^2^, n = 3,275). Although increases in BMI were small in all groups, the extent of the increase was greater in the high BMI group; the relative increase in BMI throughout the observation period was 1.0% in low, 1.2% in medium, and 2.1% in high BMI groups. All groups showed sufficiently high average posterior probability of belonging to each group ( >0.95). When we compared individuals who developed diabetes with those who did not, individuals who did not develop diabetes showed a significantly lower BMI trajectory than those who developed diabetes assessed by low, medium, and high BMI groups, respectively (all P < 0.001).

Three BMI groups among adults who developed type 2 diabetes showed distinct trajectories of visceral fat accumulation between the groups (all P < 0.001) (Fig. 1b). The mean visceral fat level 9 years before diagnosis was 164.7 cm^2^ in the high BMI group, 131.4 cm^2^ in the medium group, and 90.2 cm^2^ in the low group. The absolute increase in visceral fat over time was greatest in the high BMI group: there was a 40 cm^2^ gain in the high BMI group (relative increase of 24.1% from 9 years before diagnosis to the date of diagnosis) and 30 cm^2^ in the low and medium groups (33.6% and 27.0% increase, respectively). In contrast, the increase in mean visceral fat levels was small during the observation period for all groups that did not develop diabetes (<10 cm^2^ increase), although the trajectories of visceral fat were distinct from each other (all P < 0.001). Trajectories of visceral fat in adults who did not develop diabetes were significantly lower as compared with adults who developed diabetes, when assessed by low, medium, and high BMI groups, respectively (all P < 0.001). The linear slope and intercept for each of the trajectories are summarized in Supplementary Table S4.

Similar results to those for visceral fat were observed for subcutaneous fat (Fig. 1c). Distinct trajectories of subcutaneous fat were shown among the six groups (all P < 0.001). Abdominal subcutaneous fat level in adults who did not develop diabetes was significantly lower than that in adults who developed diabetes for a given BMI level.

Changes in the ratio of visceral to subcutaneous fat are shown in Fig. 1d. Low and medium BMI groups that developed diabetes showed a similar extent of a gradual increase in the ratio in a 10-year observation. The high BMI group that developed diabetes showed consistently lower levels of the ratio compared with that of the other two groups (all P < 0.05). As regards adults who did not develop diabetes, the trajectory of the ratio was relatively constant over the observation period, although the trajectories were distinct among the three groups (all P < 0.05).

**Figure 2:**
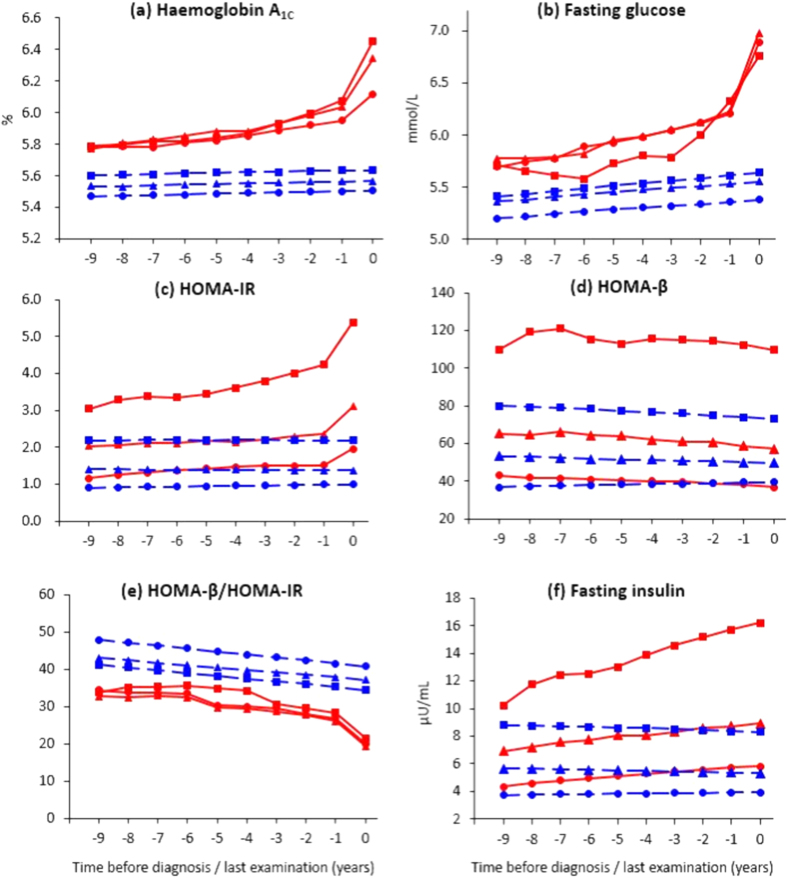
Average changes in HbA_1c_, fasting glucose, HOMA-IR, HOMA-β, ratio of HOMA-β to HOMA-IR, and fasting insulin from 9 years before diagnosis/last exam by BMI trajectory. Solid lines in red indicate estimated trajectories for the groups that developed diabetes, while dashed lines in blue indicate trajectories for each group that did not develop diabetes. Square, high BMI; triangle, medium BMI; circle, low BMI.

Figure 2a demonstrates longitudinal changes in mean HbA_1c_ levels according to BMI trajectory patterns. Among those who developed diabetes, mean levels of HbA_1c_ gradually increased over the 9-year period until 1 year before diabetes diagnosis; it then sharply increased from this point until the time of diagnosis in all three groups, although the trajectory of HbA_1c_ in the low BMI group was slightly lower than that in the medium and high BMI groups (all P < 0.001). Adults who did not develop diabetes had consistently low HbA_1c_ levels throughout the observation period, although the trajectories of HbA_1c_ were distinct among the three groups (all P < 0.05).

Similar trajectories to that for HbA_1c_ were observed for fasting glucose (Fig. 2b). The trajectory of fasting glucose prior to diabetes diagnosis in the high BMI group was significantly lower than that in the low and medium BMI groups (all P < 0.001). Individuals who did not develop diabetes had consistently low fasting plasma glucose levels, although the trajectories of fasting glucose were distinct from each other (all P < 0.001).

The three BMI groups that developed diabetes showed distinct between-group trajectories of average HOMA-IR scores (Fig. 2c, all P < 0.001). For example, when assessed 9 years before diagnosis, values were 3.0 in the high, 2.0 in the medium, and 1.2 in the low BMI groups. All BMI groups that developed diabetes showed a gradual increase in mean HOMA-IR from 9 years to 1 year before diagnosis, and then a sharp increase in HOMA-IR until the time of diagnosis. In contrast, HOMA-IR scores were stable during the observation period in the three groups that did not develop diabetes, although the trajectories of HOMA-IR were distinct from each other (all P < 0.001). The trajectory of HOMA-IR in adults who did not develop diabetes was significantly lower as compared with adults who developed diabetes, when tested according to low, medium, and high BMI groups, respectively (all P < 0.001).

Distinct trajectories of average HOMA-β scores were present among adults who developed diabetes (Fig. 2d, all P < 0.001), with values of 110.0 in the high BMI group, 65.1 in the medium group, and 43.1 in the low group 9 years before diabetes diagnosis. All six groups, except for the low BMI group that did not develop diabetes, showed a trend of decreasing HOMA-β over time. The trajectories of HOMA-β in adults who developed diabetes were significantly higher than those in adults who did not develop diabetes in medium and high BMI groups, respectively (all P < 0.001).

In all three groups that developed diabetes, ratios of HOMA-β to HOMA-IR similarly and gradually decreased from 9 years to 1 years before diagnosis, and then steeply decreased until the time of diagnosis (Fig. 2e, all P > 0.05). For example, in the high BMI group that developed diabetes, the ratio was 33.9 9 years before diagnosis and 21.5 at the time of diagnosis. In contrast to individuals who developed diabetes, the ratios were consistently high in all three groups that did not develop diabetes, although the trajectories were distinct from each other (all P < 0.001).

Trajectories for fasting insulin are shown in Fig. 2f. The three groups that developed diabetes showed distinct trajectories of fasting insulin compared with each other (all P < 0.001). For example, when assessed 9 years before diagnosis, fasting insulin concentrations were 10.2 μU/mL in the high, 6.9 μU/mL in the medium, and 4.3 μU/mL in the low BMI groups. The three groups that developed diabetes showed a gradual increase in average fasting insulin concentrations over a 10-year period. In contrast, average fasting insulin concentrations were stable during observation in the three groups that did not develop diabetes, even though the trajectories of fasting insulin were substantially distinct (all P < 0.001). The trajectory of fasting insulin in adults who did not develop diabetes was significantly lower than that in adults who developed diabetes, when examined according to low, medium, and high BMI groups, respectively (all P < 0.001).

Participants in the six groups also demonstrated distinctive characteristics at the time of first examination (Table 2). Over all, all BMI groups that developed diabetes tended to have hypertension and family history of diabetes and were more likely to smoke as compared with the groups that did not develop diabetes. Medium and high BMI groups that developed diabetes were also less likely to exercise. Higher BMI groups tended to sleep less and to work longer hours in both adults who developed and those who did not develop diabetes (all P for trend <0.01).

As shown in Table 3, at the time of diabetes diagnosis or last examination, the high BMI group that developed diabetes was younger (46.9 years) compared with the other five groups (51.6 to 53.1 years). The low BMI group that did not develop diabetes had a lower proportion of men compared with the other five groups (80.5% vs. 91.2 to 94.1%) and had a lower BMI than the low BMI group that developed diabetes. Adults who developed type 2 diabetes had significantly higher BMI levels compared with those who did not develop diabetes when assessed by low, medium, and high BMI groups, respectively.

## Discussion

This study identified three distinct BMI trajectory patterns in a cohort of Japanese adults who did and did not develop diabetes, respectively, over a 10-year observational period. BMI groups that developed diabetes demonstrated distinct levels of obesity, visceral fat, HOMA-β, and HOMA-IR among the groups, and these parameters worsened until diabetes diagnosis. Although BMI groups of the individuals who did not develop diabetes also showed distinct levels of these variables across groups, the levels were materially unchanged over time in all groups. These data could contribute to the understanding of type 2 diabetes pathogenesis in the Asian population.

Previous studies have demonstrated that Asians develop type 2 diabetes at a lower degree of obesity compared with Caucasians^3^. However, heterogeneity in the development of obesity before diabetes onset in Asians is poorly understood. Among Caucasians in the UK, it was shown that nearly all adults who developed diabetes were classified into a BMI group showing a trajectory pattern termed “stable overweight” with gradual increases in BMI (0.13 kg/m^2^ per year) over an 18-year observation period^9^. Other individuals who developed diabetes were termed “progressive weight gainers”, who had a 7.6 kg/m^2^ weight increase 4–5 years before diagnosis, or “persistently obese”^9^. All the groups were characterized by a consistently high BMI: mean 27.0 kg/m^2^ in the “stable overweight” group, 34.2 kg/m^2^ in “progressive weight gain,” and 38.2 kg/m^2^ in “persistently obese” at the first examination^9^. Our findings among the Japanese population differ in the distribution of BMI at a single point. We found that more than one-third of adults who developed diabetes had a BMI trajectory characterized by low-normal weight (21.9 kg/m^2^, 9 years before onset), a pattern not observed among Caucasians. The medium BMI trajectory among those who developed diabetes in our study was similar to the “stable overweight” group in the Caucasian population^9^, although the proportion of this group in the present study was comparatively smaller. Our finding of a gradual increase in BMI before diabetes onset in this group is consistent with the “stable overweight” pattern^9^.

High BMI itself and weight increases over time have been independently associated with increased diabetes risk^21^. However, longitudinal patterns of changes in BMI over time in adults who have and have not developed diabetes are scarce. Although a recent Japanese study^4^ described BMI trajectory before diabetes onset, heterogeneity in the trajectory was not investigated. In contrast, we identified distinct BMI trajectory patterns before diabetes diagnosis among Asians. We also found that individuals who developed diabetes had higher BMIs in all groups compared with those who did not develop diabetes. Notably, the magnitude of weight increase over time in individuals who developed diabetes was greater than that of those who did not. Our data suggest that preventing weight gain and/or reducing body weight may be important to reduce diabetes risk irrespective of obesity level.

East Asians have much lower β-cell function than Caucasians, and thus are more vulnerable to increases in insulin resistance^22^. A recent Korean study suggested that impaired β-cell compensation for insulin resistance over time is critical in type 2 diabetes development^23^. However, this study did not investigate trajectory heterogeneity. We elucidated that the ratio of HOMA-β to HOMA-IR, an indicator of impaired β-cell compensation for insulin resistance, gradually decreased from 9 years to 1 year before diabetes onset, and then sharply decreased until onset in all trajectory patterns. These findings suggest that declining β-cell compensation for insulin resistance may contribute to type 2 diabetes onset regardless of obesity levels. Moreover, visceral fat accumulation has been suggested to explain ethnic differences in insulin resistance and diabetes^24^. Although a study among Japanese Americans showed that individuals who developed type 2 diabetes had greater increases in visceral fat before diabetes onset^25^, this study did not investigate the changes within subgroups. We demonstrated that both visceral and subcutaneous fat markedly increased over time in all groups developing diabetes, whereas fat measures only increased slightly among the groups that did not. Interestingly, among the low and medium BMI groups that developed diabetes, visceral fat increased more than subcutaneous fat, as indicated by the ratio of visceral to subcutaneous fat. This finding suggests that visceral fat accumulation plays a dominant role in diabetes development among non-obese Japanese adults. Our finding is supported by a prospective study of Japanese Americans, which reported a greater impact of visceral fat compared with subcutaneous fat on diabetes development^26^. Accumulation of visceral fat over time may enhance insulin resistance, possibly through dysregulation of adipokines released from visceral fat^27^, resulting in diabetes.

A strength of this study included a large sample size, with sufficient numbers of incident diabetes cases to detect BMI trajectory patterns. Additionally, this is the first study to report visceral and subcutaneous fat trajectories with a large sample size. However, several limitations should be mentioned. Firstly, generalizability of the study may be limited due to the specific population analysed. The majority of participants were young to middle-aged Japanese male workers. Caution should therefore be taken in generalizing our findings to other ethnicities, the general population, women, or the elderly. Another limitation was the limited availability of data on visceral and subcutaneous fat, and fasting insulin (Supplementary Table S1). Nonetheless, age and BMI levels between those who have visceral adiposity data and those who did not have were not largely different within the trajectory group (Supplementary Table S3). Further, our data on visceral and subcutaneous fat^28^, insulin resistance^29^, and β-cell function^29^ are comparable to previous Japanese studies. Additionally, our finding of low ratio of visceral fat to subcutaneous fat (more subcutaneous fat accumulation) in obese adults are consistent with previous findings^30^.

In conclusion, we identified three distinct BMI trajectories prior to the onset of type 2 diabetes among Japanese workers during a 10-year observation, all showing gradual weight gain. Our data indicate that weight gain due to visceral fat accumulation induces β-cell failure in compensation for insulin resistance, leading to type 2 diabetes onset regardless of obesity levels in the Japanese population.

## Additional Information

**How to cite this article:** Kuwahara, K. *et al*. Body mass index trajectory patterns and changes in visceral fat and glucose metabolism before the onset of type 2 diabetes. *Sci. Rep.* **7**, 43521; doi: 10.1038/srep43521 (2017).

**Publisher's note:** Springer Nature remains neutral with regard to jurisdictional claims in published maps and institutional affiliations.

## Supplementary Material

Supporting Information

## Figures and Tables

**Table 1:** Estimated parameters of body mass index trajectories among adults who did and did not develop diabetes.

BMI trajectories	Intercept, kg/m^2^	Linear slope, kg/m^2^	Group	Average group
	Estimate (95% CI)	Estimate (95% CI)	proportion, n (%)	probability
**Developed diabetes**				
Low BMI group	21.5 (21.4, 21.7)	0.09 (0.07, 0.11)	728 (38.5)	0.97
Medium BMI group	25.4 (25.3, 25.4)	0.14 (0.13, 0.16)	898 (47.5)	0.96
High BMI group	30.1 (29.8, 30.5)	0.30 (0.24, 0.35)	266 (14.1)	0.98
**Diabetes not developed**				
Low BMI group	20.3 (20.3, 20.3)	0.02 (0.02, 0.03)	7,887 (35.7)	0.97
Medium BMI group	23.8 (23.8, 23.8)	0.03 (0.03, 0.03)	10,924 (49.5)	0.97
High BMI group	28.0 (28.0, 28.1)	0.07 (0.06, 0.08)	3,275 (14.8)	0.98

Abbreviations: BMI, body mass index; CI, confidence interval.

Data on intercept and slope were obtained using linear regression analysis.

**Table 2:** Characteristics of adults who did and did not develop type 2 diabetes at the time of first health examination (April 2006 to March 2007), according to body mass index trajectory patterns.

	Developed diabetes			P-trend^a^	Diabetes not developed			P-trend
	Low BMI	Medium BMI	High BMI		Low BMI	Medium BMI	High BMI	
Number of participants	728 (38.5)	898 (47.5)	266 (14.1)		7,887 (35.7)	10,924 (49.5)	3,275 (14.8)	
Age, year	48.4 ± 7.1	46.8 ± 7.3	42.0 ± 6.8	<0.001	44.6 ± 8.2	45.6 ± 8.1	44.2 ± 7.7	0.68
Sex, % men	684 (94.0)	845 (94.1)	245 (92.1)	0.42	6,345 (80.5)	10,082 (92.3)	2,998 (91.2)	<0.001
BMI, kg/m^2^	21.9 ± 1.8	26.0 ± 1.5	31.4 ± 2.8	<0.001	20.3 ± 1.5	23.8 ± 1.5	28.1 ± 2.3	<0.001
Visceral fat, cm^2^	106 ± 47	152 ± 44	195 ± 54	<0.001	78 ± 42	118 ± 42	157 ± 46	<0.001
Subcutaneous fat, cm^2^	102 ± 35	158 ± 40	283 ± 89	<0.001	92 ± 36	135 ± 38	203 ± 60	<0.001
Visceral to subcutaneous fat ratio	1.0 ± 0.4	1.0 ± 0.4	0.8 ± 0.3	0.001	0.9 ± 0.4	0.9 ± 0.3	0.8 ± 0.3	0.12
Hypertension, %	166 (22.8)	289 (32.2)	111 (41.7)	<0.001	804 (10.2)	1,844 (16.9)	901 (27.5)	<0.001
Fasting glucose, mmol/L	5.9 ± 0.5	5.9 ± 0.5	5.7 ± 0.6	<0.001	5.2 ± 0.5	5.4 ± 0.5	5.5 ± 0.5	<0.001
HbA_1c_, %	5.8 ± 0.3	5.9 ± 0.3	5.9 ± 0.3	0.21	5.4 ± 0.3	5.5 ± 0.3	5.6 ± 0.3	<0.001
Fasting insulin, μU/mL	5.3 ± 2.9	8.7 ± 4.0	15.6 ± 6.7	<0.001	4.4 ± 2.3	6.2 ± 3.2	9.3 ± 4.7	<0.001
HOMA-IR	1.4 ± 0.8	2.4 ± 1.1	4.1 ± 1.7	<0.001	1.1 ± 0.6	1.6 ± 0.8	2.3 ± 1.2	<0.001
HOMA-β	40.9 ± 22.0	68.9 ± 35.2	135.5 ± 78.6	<0.001	44.5 ± 23.1	59.0 ± 30.7	88.2 ± 45.8	<0.001
HOMA-β/HOMA-IR	30.3 ± 9.5	30.2 ± 9.4	32.8 ± 11.4	0.35	43.9 ± 13.6	40.0 ± 11.9	39.0 ± 9.8	<0.001
Family history of diabetes, %	174 (23.9)	205 (22.8)	54 (20.3)	0.25	989 (12.5)	1,433 (13.1)	449 (13.7)	0.08
Smoking, %	359 (49.3)	415 (46.2)	130 (48.9)	0.58	3,231 (41.0)	4,569 (41.8)	1,431 (43.7)	0.01
Heavy alcohol use, %^b^	100 (13.7)	99 (11.0)	18 (6.8)	0.002	778 (9.9)	1,134 (10.4)	283 (8.6)	0.23
Sleeping <6 h/d, %	334 (45.9)	474 (52.8)	175 (65.8)	<0.001	3,812 (48.3)	5,583 (51.1)	1,848 (56.4)	<0.001
Low leisure exercise, %^c^	598 (82.1)	755 (84.1)	226 (85.0)	0.22	6,552 (83.1)	8,785 (80.4)	2,648 (80.9)	<0.001
Sedentary work, %	435 (59.8)	561 (62.5)	178 (66.9)	0.04	4,596 (58.3)	6,880 (63.0)	2,083 (63.6)	<0.001
Long overtime work, %^d^	232 (31.9)	377 (42.0)	98 (36.8)	0.007	2,704 (34.3)	4,302 (39.4)	1,311 (40.0)	<0.001
Shift work, %	107 (14.7)	155 (17.3)	57 (21.4)	0.012	1,413 (17.9)	1,883 (17.2)	660 (20.2)	0.059
Low commuting activity, %^e^	352 (48.4)	441 (49.1)	143 (53.8)	0.19	4,259 (54.0)	5,738 (52.5)	1,788 (54.6)	0.84

^a^P values for trend was calculated using linear regression for continuous variables and logistic regression for categorical variables, respectively

^b^≥2 *go* of Japanese sake equivalent per day.

^c^<7.5 metabolic equivalent hours per week.

^d^≥45 hours of overtime per month.

^e^<20 min of walking for commuting to and from work.

Abbreviations: BMI, body mass index; HOMA-IR; homeostasis model assessment for insulin resistance; HOMA-β, homeostasis model assessment for β cell function. Data are shown as mean ± standard deviation for continuous variables and number (percentage) for categorical variables. Data on visceral adiposity, fasting insulin, and HOMAs are available among subgroups; numbers (percentage) in each subcategory are shown in Supplementary Table S1.

**Table 3:** Characteristics of adults who did and did not develop type 2 diabetes at the time of the last health examination according to body mass index trajectory patterns.

	Developed diabetes			Diabetes not developed		
	Low BMI	Medium BMI	High BMI	Low BMI	Medium BMI	High BMI
Number of participants	728 (38.5)	898 (47.5)	266 (14.1)	7,887 (35.7)	10,924 (49.5)	3,275 (14.8)
Age, year	53.1 ± 7.0	51.5 ± 7.2	46.9 ± 7.1	51.9 ± 7.6	52.8 ± 7.4	51.6 ± 7.2
Sex, % men	684 (94.0)	845 (94.1)	245 (92.1)	6,346 (80.5)	10,082 (92.3)	2,988 (91.2)
BMI, kg/m^2^	22.4 ± 1.9	26.8 ± 1.7	33.1 ± 3.1	20.5 ± 1.5	24.1 ± 1.4	28.6 ± 2.5

Abbreviations: BMI, body mass index.

Data are shown as mean ± SD for continuous variables and number (percentage) for categorical variables.
