# Supplementary material for: Body mass index trajectory patterns and changes in visceral fat and glucose metabolism before the onset of type 2 diabetes
Source: Sci Rep. 2017 Mar 7;7:43521. doi: 10.1038/srep43521 (PMC5339907; doi:10.1038/srep43521)
Supplement: Supporting Information [file srep43521-s1.pdf]

SUPPORTING INFORMATION

Body mass index trajectory patterns and changes in visceral fat and glucose metabolism before the onset of type 2 diabetes

Keisuke Kuwahara[1,2,*], Toru Honda[3], Tohru Nakagawa[3], Shuichiro Yamamoto[3], Takeshi Hayashi[3], Tetsuya Mizoue[1]

Table of contents: **Page No.**

Supplementary Figure S1……………………………………………..S2
Supplementary Figure S2……………………………………………..S3

Supplementary Table S1………………………………………………S4-S6
Supplementary Table S2………………………………………………S7
Supplementary Table S3………………………………………………S8
Supplementary Table S4………………………………………………S9-S12

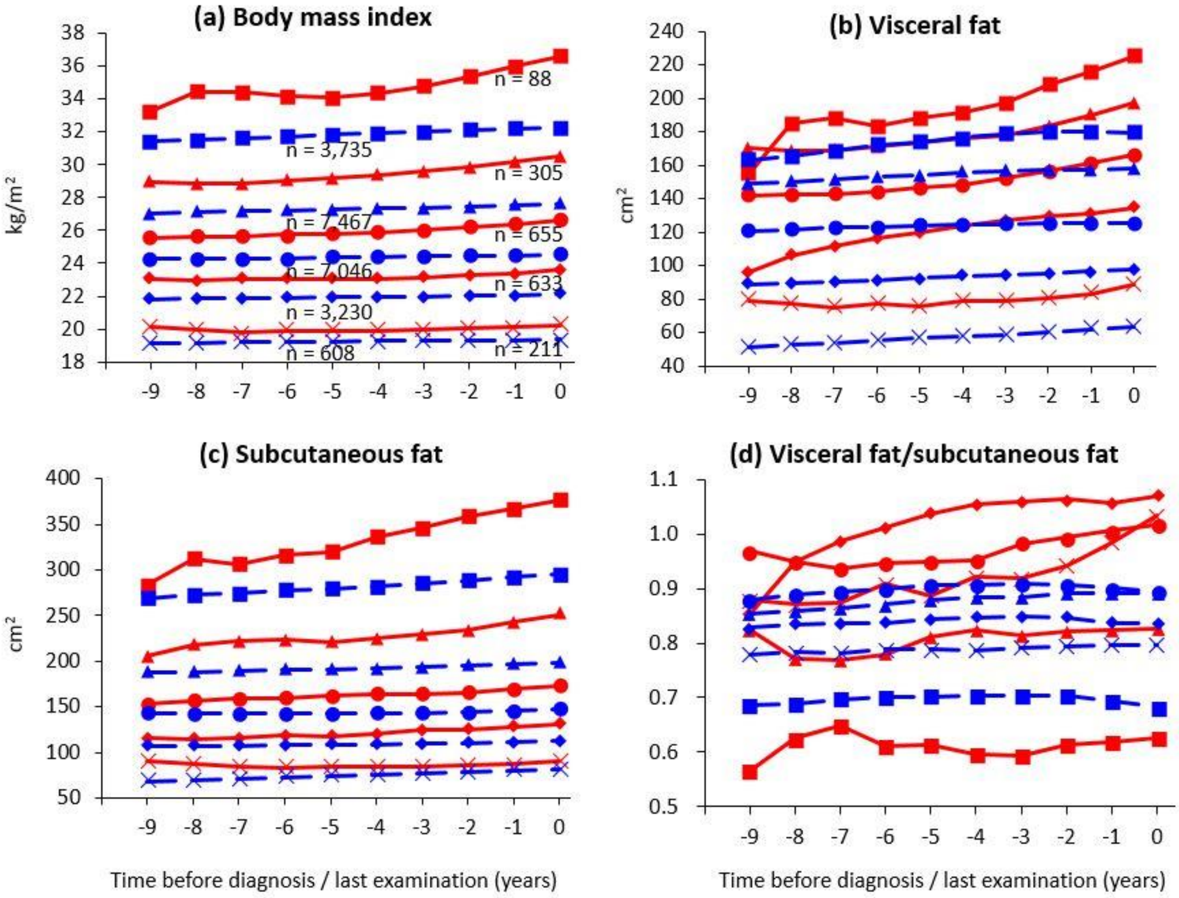

Supplementary Figure S1: Average changes in BMI, visceral and subcutaneous fat, and ratio of visceral to subcutaneous fat from 9 years before diagnosis/last exam by five BMI groups in adults who developed diabetes (red) and adults who did not develop diabetes (blue)

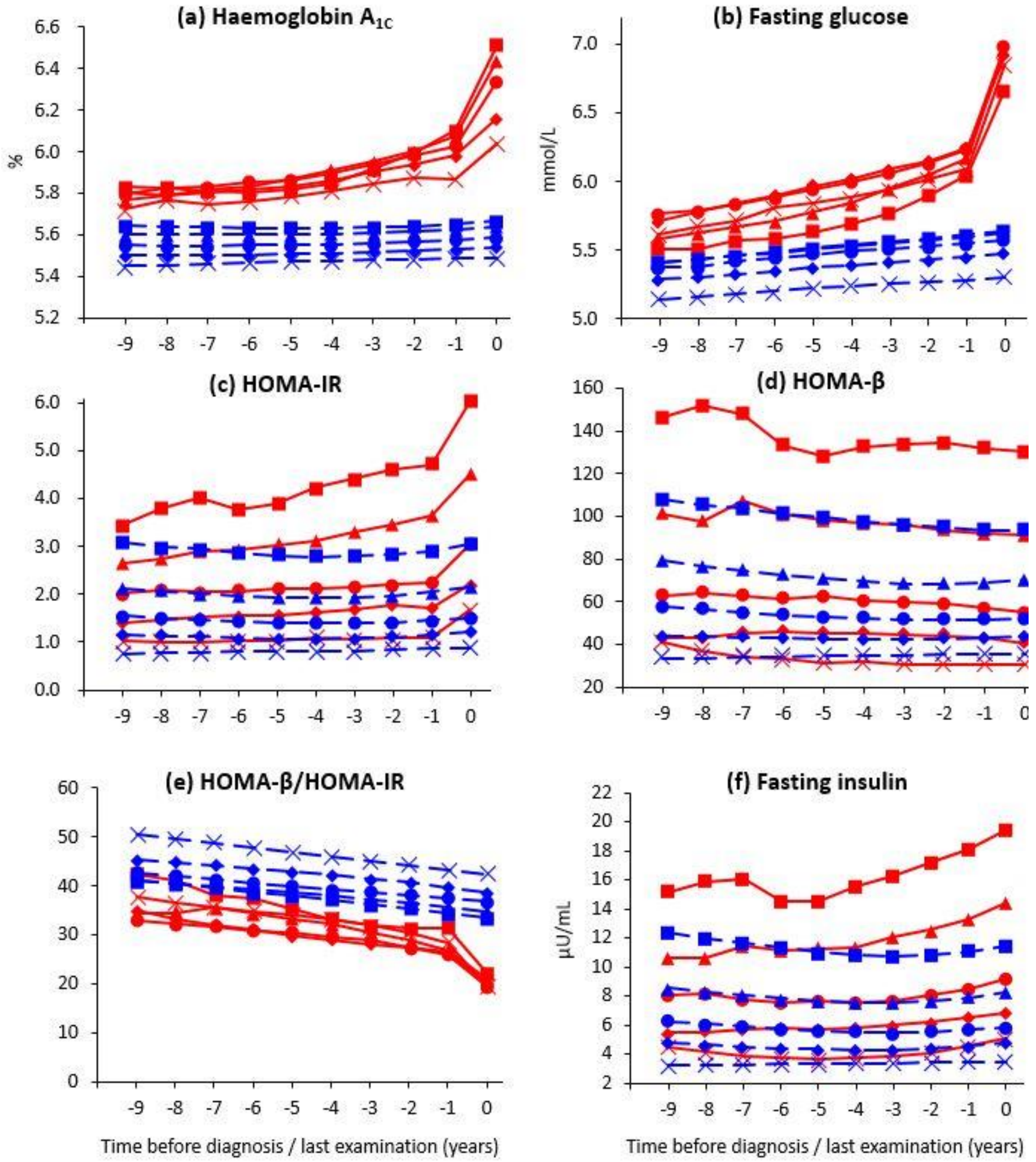

Supplementary Figure S2: Average changes in HbA_{1c}, fasting glucose, HOMA-IR, HOMA-β, ratio of HOMA-β to HOMA-IR, and fasting insulin from 9 years before diagnosis/last exam by five BMI groups in adults who developed diabetes (red) and adults who did not develop diabetes (blue)

Supplementary Table S1: The number of participants with data on body mass index (BMI), glucose metabolism, and visceral fat according to BMI trajectory

	**Time before diagnosis or last examination**									
	9 years	8 years	7 years	6 years	5 years	4 years	3 years	2 years	1 year	0 year
**BMI**										
Developed diabetes										
Low BMI (n = 728)	79 (10.9)	168 (23.1)	220 (30.2)	290 (39.8)	425 (58.4)	519 (71.3)	620 (85.2)	700 (96.2)	692 (95.1)	728 (100)
Medium BMI (n = 898)	102 (11.4)	202 (22.5)	293 (32.6)	362 (40.3)	485 (54.0)	616 (68.6)	742 (82.6)	866 (96.4)	859 (95.7)	898 (100)
High BMI (n = 266)	33 (12.4)	60 (22.6)	86 (32.3)	119 (44.7)	156 (58.6)	192 (72.2)	224 (84.2)	260 (97.7)	258 (97.0)	266 (100)
Diabetes not developed										
Low BMI (n = 7,887)	5,362 (69.7)	5,731 (74.5)	6,020 (78.0)	6,420 (82.7)	6,699 (86.0)	6,998 (89.7)	7,277 (92.9)	7,596 (96.6)	7,603 (96.6)	7,887 (100)
Medium BMI (n = 10,924)	7,417 (68.5)	7,889 (72.7)	8,284 (76.3)	8,888 (81.6)	9,285 (84.8)	9,686 (88.4)	10,030 (91.5)	10,481 (95.7)	10,510 (96.0)	10,924 (100)
High BMI (n = 3,275)	2,363 (66.7)	2,7464 (70.3)	2,606 (74.9)	2,764 (80.9)	2,849 (85.3)	2,974 (89.7)	3,067 (93.3)	3,160 (96.8)	3,166 (96.9)	3,275 (100)
**Fasting glucose**										
Developed diabetes										
Low BMI	79 (10.9)	168 (23.1)	220 (30.2)	290 (39.8)	425 (58.4)	519 (71.3)	620 (85.2)	700 (96.2)	692 (95.1)	728 (100)
Medium BMI	102 (11.4)	202 (22.5)	293 (32.6)	362 (40.3)	485 (54.0)	616 (68.6)	742 (82.6)	866 (96.4)	859 (95.7)	898 (100)
High BMI	33 (12.4)	60 (22.6)	86 (32.3)	119 (44.7)	156 (58.6)	192 (72.2)	224 (84.2)	260 (97.7)	258 (97.0)	266 (100)
Diabetes not developed										
Low BMI	5,362 (68.0)	5,731 (72.7)	6,020 (76.3)	6,420 (81.4)	6,699 (84.9)	6,998 (88.7)	7,277 (92.3)	7,596 (96.3)	7,603 (96.4)	7,887 (100)
Medium BMI	7,417 (67.9)	7,889 (72.2)	8,284 (75.8)	8,888 (81.4)	9,285 (85.0)	9,686 (88.7)	10,030 (91.8)	10,481 (95.9)	10,510 (96.2)	10,924 (100)
High BMI	2,363 (72.2)	2,464 (75.2)	2,606 (79.6)	2,761 (84.3)	2,849 (87.0)	2,974 (90.8)	3,067 (93.6)	3,160 (96.5)	3,166 (96.7)	3,275 (100)
**HbA_{1c}**										
Developed diabetes										

Low BMI	79 (10.9)	168 (23.1)	220 (30.2)	290 (39.8)	425 (58.4)	519 (71.3)	620 (85.2)	700 (96.2)	692 (95.1)	728 (100)
Medium BMI	102 (11.4)	202 (22.5)	293 (32.6)	362 (40.3)	485 (54.0)	616 (68.6)	742 (82.6)	866 (96.4)	859 (95.7)	898 (100)
High BMI	33 (12.4)	60 (22.6)	86 (32.3)	119 (44.7)	156 (58.6)	192 (72.2)	224 (84.2)	260 (97.7)	258 (97.0)	266 (100)
Diabetes not developed										
Low BMI	5,362 (68.0)	5,731 (72.7)	6,020 (76.3)	6,420 (81.4)	6,699 (84.9)	6,998 (88.7)	7,277 (92.3)	7,596 (96.3)	7,603 (96.4)	7,887 (100)
Medium BMI	7,417 (67.9)	7,889 (72.2)	8,284 (75.8)	8,888 (81.4)	9,285 (85.0)	9,686 (88.7)	10,030 (91.8)	10,481 (95.9)	10,510 (96.2)	10,924 (100)
High BMI	2,363 (72.2)	2,464 (75.2)	2,606 (79.6)	2,761 (84.3)	2,849 (87.0)	2,974 (90.8)	3,067 (93.6)	3,160 (96.5)	3,166 (96.7)	3,275 (100)
**Fasting insulin**										
Developed diabetes										
Low BMI	47 (6.5)	89 (12.2)	113 (15.5)	142 (19.5)	201 (27.6)	238 (32.7)	287 (39.4)	299 (41.1)	292 (40.1)	313 (43.0)
Medium BMI	51 (5.7)	107 (11.9)	144 (16.0)	176 (19.6)	235 (26.2)	286 (31.8)	343 (38.2)	390 (43.4)	389 (43.3)	404 (45.0)
High BMI	12 (4.5)	29 (10.9)	41 (15.4)	55 (20.7)	67 (25.2)	85 (32.0)	101 (38.0)	113 (42.5)	112 (42.1)	113 (42.5)
Diabetes not developed										
Low BMI	1,911 (24.2)	2,020 (25.6)	2,131 (27.0)	2,293 (29.1)	2,380 (30.2)	2,452 (31.1)	2,482 (31.5)	2,512 (31.8)	2,505 (31.8)	2,617 (33.2)
Medium BMI	3,277 (30.0)	3,454 (31.6)	3,617 (33.1)	3,888 (35.6)	4,104 (37.6)	4,251 (38.9)	4,285 (39.2)	4,331 (39.6)	4,339 (39.7)	4,530 (41.5)
High BMI	1,036 (31.6)	1,074 (32.8)	1,129 (34.5)	1,210 (36.9)	1,243 (38.0)	1,281 (39.1)	1,298 (39.6)	1,307 (39.9)	1,308 (39.9)	1,351 (41.3)
**Visceral fat**										
Developed diabetes										
Low BMI	39 (5.4)	78 (10.7)	100 (13.7)	123 (16.9)	177 (24.3)	202 (27.7)	242 (33.2)	254 (34.9)	247 (33.9)	267 (36.7)
Medium BMI	43 (4.8)	92 (10.2)	126 (14.0)	151 (16.8)	202 (22.5)	241 (26.8)	288 (32.1)	329 (36.6)	331 (36.9)	343 (38.2)
High BMI	12 (4.5)	27 (10.2)	37 (13.9)	47 (17.7)	58 (21.8)	70 (26.3)	84 (31.6)	96 (36.1)	95 (35.7)	96 (36.1)
Diabetes not developed										
Low BMI	1,511 (19.2)	1,607 (20.4)	1,697 (21.5)	1,831 (23.2)	1,897 (24.1)	1,958 (24.8)	1,976 (25.1)	2,004 (25.4)	2,002 (25.4)	2,100 (26.6)
Medium BMI	2,728 (25.0)	2,886 (26.4)	3,029 (27.7)	3,259 (29.8)	3,445 (31.5)	3,574 (32.7)	3,599 (32.9)	3,648 (33.4)	3,651 (33.4)	3,829 (35.1)

High BMI	885 (27.0)	922 (28.2)	971 (29.6)	1,034 (31.6)	1,069 (32.6)	1,100 (33.6)	1,114 (34.0)	1,124 (34.3)	1,123 (34.3)	1,163 (35.5)

Abbreviations: BMI, body mass index.

Data are shown as n (%). Numbers (%) for homeostasis model assessment are the same as those for fasting insulin. N (%) for subcutaneous fat are the same as those for visceral fat.

Supplementary Table S2: Characteristics at the first examination (April 2006-March 2007) among excluded and included participants

	Excluded	Included
n	18,351	23,978
Men, %	14,189 (77.3)	21,189 (88.4)
Age (year)	43.7 ± 10.4	45.2 ± 8.1
BMI (kg/m^2)	23.6 ± 3.7	23.4 ± 3.1

Data are shown as mean ± SD or n (%).

Supplementary Table S3: Characteristics of participants at the time of diabetes diagnosis or last examination by BMI groups between individuals who had data on visceral fat during observation and those who had no data on visceral fat

	n	Age (year)	Sex, %	BMI (kg/m^2)
Developed diabetes				
Low BMI				
Having visceral fat data	267	53.9 ± 6.8	263 (98.5)	22.6 ± 1.7
Not having data	461	52.7 ± 7.1	421 (91.3)	22.3 ± 1.9
Medium BMI				
Having visceral fat data	343	52.5 ± 7.1	339 (98.8)	26.8 ± 1.6
Not having data	555	50.9 ± 7.2	506 (91.2)	26.8 ± 1.7
High BMI				
Having visceral fat data	96	47.3 ± 6.8	96 (100)	33.0 ± 3.2
Not having data	170	46.7 ± 7.2	149 (87.7)	33.2 ± 3.1
Not developed diabetes				
Low BMI				
Having visceral fat data	2,100	53.4 ± 7.1	1,946 (92.7)	20.8 ± 1.4
Not having data	5,787	51.3 ± 7.7	4,400 (76.0)	20.4 ± 1.6
Medium BMI				
Having visceral fat data	3,829	54.0 ± 6.9	3,749 (97.9)	24.1 ± 1.4
Not having data	7,095	52.2 ± 7.6	6,333 (89.3)	24.1 ± 1.4
High BMI				
Having visceral fat data	1,163	52.8 ± 6.8	1,132 (97.3)	28.5 ± 2.3
Not having data	2,112	50.9 ± 7.3	1,856 (87.9)	28.7 ± 2.6

Data are shown as mean ± SD or number (%).

Supplementary Table S4: Estimated trajectories of visceral adiposity and glucose metabolism markers assessed for body mass index groups among adults who did and did not develop diabetes

	**Intercept (95% CI)**	**Linear slope (95% CI)**
**Visceral fat (cm^2)**		
Developed diabetes		
Low BMI group	93.6 (88.7, 98.5)	2.99 (2.21, 3.77)
Medium BMI group	131.8 (128.0, 135.6)	3.75 (3.15, 4.35)
High BMI group	165.4 (158.3, 172.4)	4.15 (3.04, 5.26)
Diabetes not developed		
Low BMI	70.5 (69.6, 71.4)	0.95 (0.78, 1.11)
Medium BMI group	115.4 (114.7, 116.2)	0.65 (0.52, 0.78)
High BMI group	153.8 (152.4, 155.2)	1.21 (0.95, 1.46)
**Subcutaneous fat (cm^2)**		
Developed diabetes		
Low BMI group	98.2 (94.4, 102.0)	1.93 (1.33, 2.53)
Medium BMI group	152.9 (148.7, 157.1)	2.56 (1.90, 3.22)
High BMI group	241.0 (227.5, 254.6)	6.39 (4.25, 8.53)
Diabetes not developed		
Low BMI	87.6 (86.7, 88.5)	0.81 (0.65, 0.96)
Medium BMI group	135.4 (134.7, 136.1)	0.45 (0.32, 0.58)
High BMI group	204.3 (202.2, 206.4)	1.34 (0.96, 1.73)
**Visceral/subcutaneous fat**		
Developed diabetes		
Low BMI group	0.9 (0.9, 1.0)	0.02 (0.01, 0.02)

Medium BMI group	0.9 (0.9, 0.9)	0.01 (0.006, 0.02)
High BMI group	0.7 (0.7, 0.7)	0.003 (-0.004, 0.01)
Diabetes not developed		
Low BMI	0.8 (0.8, 0.8)	0.003 (0.002, 0.005)
Medium BMI group	0.9 (0.9, 0.9)	0.002 (0.001, 0.004)
High BMI group	0.8 (0.8, 0.8)	0.001 (-0.001, 0.003)
**HbA_{1c} (%)**		
Developed diabetes		
Low BMI group	5.7 (5.7, 5.7)	0.04 (0.04, 0.04)
Medium BMI group	5.6 (5.6, 5.7)	0.06 (0.06, 0.06)
High BMI group	5.6 (5.5, 5.6)	0.07 (0.07, 0.08)
Diabetes not developed		
Low BMI	5.5 (5.5, 5.5)	0.004 (0.003, 0.005)
Medium BMI group	5.5 (5.5, 5.5)	0.004 (0.004, 0.005)
High BMI group	5.6 (5.6, 5.6)	0.004 (0.003, 0.005)
**Fasting plasma glucose (mmol/L)**		
Developed diabetes		
Low BMI group	5.4 (5.4, 5.5)	2.16 (2.08, 2.25)
Medium BMI group	5.4 (5.4, 5.5)	2.30 (2.22, 2.38)
High BMI group	5.2 (5.1, 5.3)	2.51 (2.36, 2.66)
Diabetes not developed		
Low BMI	5.2 (5.2, 5.2)	0.35 (0.33, 0.36)
Medium BMI group	5.4 (5.4, 5.4)	0·38 (0.37, 0.40)
High BMI group	5.4 (5.4, 5.4)	0.45 (0.43, 0.48)

**Fasting insulin (μU/mL)**		
Developed diabetes		
Low BMI group	4.5 (4.2, 4.7)	0.16 (0.11, 0.20)
Medium BMI group	7.1 (6.8, 7.4)	0.20 (0.15, 0.26)
High BMI group	10.9 (9.9, 11.8)	0.60 (0.45, 0.75)
Diabetes not developed		
Low BMI	3.7 (3.7, 3.8)	0.02 (0.02, 0.03)
Medium BMI group	5.7 (5.6, 5.7)	-0.04 (-0.04, -0.03)
High BMI group	8.8 (8.7, 8.9)	-0.06 (-0.08, -0.03)
**HOMA-IR**		
Developed diabetes		
Low BMI group	1.1 (1.0, 1.2)	0.07 (0.06, 0.08)
Medium BMI group	1.8 (1.7, 1.8)	0.10 (0.09, 0.12)
High BMI group	2.6 (2.4, 2.8)	0.24 (0.20, 0.28)
Diabetes not developed		
Low BMI	0.9 (0.9, 0.9)	0.01 (0.01, 0.01)
Medium BMI group	1.4 (1.4, 1.4)	-0.003 (-0.005, -0.001)
High BMI group	2.2 (2.2, 2.2)	-0.01 (-0.01, 0.004)
**HOMA-β**		
Developed diabetes		
Low BMI group	43.0 (40.9, 45.1)	-0.63 (-0.96, -0.30)
Medium BMI group	67.3 (64.5, 70.2)	-1.05 (-1.51, -0.60)
High BMI group	119.1 (109.2, 129.1)	-0.87 (-2.43, 0.70)
Diabetes not developed		

Low BMI	36.9 (36.6, 37.3)	0.28 (0.23, 0.34)
Medium BMI group	53.2 (52.9, 53.6)	-0.40 (-0.46, -0.34)
High BMI group	80.4 (79.2, 81.5)	-0.78 (-0.99, -0.58)
**HOMA -β/HOMA-IR**		
Developed diabetes		
Low BMI group	37.3 (36.8, 37.8)	-1.53 (-1.61, -1.46)
Medium BMI group	36.3 (35.8, 36.8)	-1.48 (-1.56, -1.40)
High BMI group	40.4 (39.2, 41.5)	-1.68 (-1.87, -1.50)
Diabetes not developed		
Low BMI	48.0 (48.9, 48.1)	-0.79 (-0.81, -0.78)
Medium BMI group	43.1 (43.0, 43.3)	-0.65 (-0.68, -0.62)
High BMI group	41.3 (41.0, 41.5)	-0.74 (-0.78, -0.70)

Abbreviations: BMI, body mass index; CI, confidence interval; HOMA-IR; homeostasis model assessment for insulin resistance; HOMA-β, homeostasis model assessment for β cell function.

Data on intercept and slope were obtained using linear regression analysis.
